# Unmasking the Hidden Threat of Cardiac Involvement in Dengue Fever: A Critical Longitudinal Study

**DOI:** 10.7759/cureus.45961

**Published:** 2023-09-25

**Authors:** Naresh Jadav, Nital Vaghela, Neha Verma, Dilan Davis, Ashish A Sonani, Piyush Patoliya

**Affiliations:** 1 Internal Medicine, Government Medical College, Surat, Surat, IND; 2 Internal Medicine, New Civil Hospital, Surat, IND; 3 Pulmonary Medicine, New Civil Hospital, Surat, IND

**Keywords:** longitudinal study, timely intervention, intensive care, cardiac involvement, dengue fever

## Abstract

Introduction: Dengue fever (DF) arises from the dengue virus (DENV), a common viral illness transmitted by arthropods. This medical condition has the potential to result in severe complications, including but not limited to liver failure, disseminated intravascular coagulation, dengue encephalopathy, myocarditis, acute renal failure, and hemolytic uremic syndrome. Evaluating cardiac manifestations in dengue is crucial for timely intervention and intensive care to save patients' lives.

Materials and methods: A longitudinal study involved 104 dengue fever patients admitted to the Department of Medicine at New Civil Hospital, Surat, between May 2021 and October 2021, to identify potential cardiac involvement.

Results: The study found that out of the 104 patients, 28 (26.92%) showed cardiac involvement based on clinical manifestations. Among these patients, 28 (26.92%) exhibited abnormal ECG results, and 39 (37.50%) showed elevated creatine kinase-MB (CK-MB) levels. Of the 28 patients who showed ECG changes, 14 (50%) displayed abnormal 2D-echocardiography (ECHO) results. The most common electrocardiographic anomaly was a T-wave inversion in V1-V4. The predominant 2D-ECHO finding was mild pericardial effusion.

Conclusion: Cardiac involvement in dengue presents atypically and can lead to conditions like myocarditis, arrhythmias, cardiac failure, or shock. Assessing cardiac manifestations in dengue patients is pivotal for appropriate management.

## Introduction

Dengue fever (DF) is the most widespread arthropod-borne viral disease, impacting millions of individuals globally annually. Caused by the dengue virus (DENV), the spectrum of the illness ranges from mild flu-like symptoms to more severe manifestations such as dengue hemorrhagic fever (DHF) and dengue shock syndrome (DSS). The complications arising from severe dengue infection, including liver failure, disseminated intravascular coagulation, dengue encephalopathy, acute renal failure, and hemolytic uremic syndrome, present substantial risks to patients' overall health [1].

Traditionally, the pathogenesis of dengue fever has been linked to capillary leakage, leading to decreased intravascular volume and the development of DHF/DSS. However, recent studies have highlighted the potential involvement of the heart in dengue-related shock [2-5]. Authors have reported cases of direct cardiac involvement in dengue fever patients, suggesting a plausible connection between cardiac dysfunction and the development of shock [3,6,7].

Despite the vital role of cardiac involvement in the pathogenesis of severe dengue, limited data exist regarding myocardial impairment in dengue fever [8]. Most previous investigations have focused on left ventricular systolic function, highlighting acute reversible myocarditis in some cases. Biomarkers, electrocardiography (ECG), and two-dimensional echocardiography (2D-ECHO) have been utilized to assess cardiac injury in dengue patients, revealing notable ST-segment and T-wave changes in ECG, as well as low ejection fractions and global hypokinesia on radionuclide ventriculography [9,10].

Recognizing this existing gap in knowledge, our current study aims to comprehensively assess the cardiac manifestations present in patients diagnosed with both dengue and DHF. By shedding light on the extent of cardiac involvement in these patients, we aim to facilitate timely and intensive management and critical care, thus potentially saving lives and improving patient outcomes.

Clinical features, electrocardiographic abnormalities, abnormal cardiac biomarkers, and echocardiographic findings have been evaluated in our study as possible markers of cardiac involvement in dengue.

Understanding the cardiac implications of dengue fever can significantly impact clinical management, especially in cases of DHF/DSS, where prompt and appropriate fluid management is crucial. This study emphasizes the importance of monitoring cardiac function in dengue patients, enabling early intervention, and providing essential insights into preventing and managing severe complications, ultimately improving patient outcomes and saving lives.

Aim and objectives

This study aims to investigate the prevalence and clinical significance of cardiac involvement in individuals afflicted by DF. The objective is to improve the identification and treatment in the early stages, ultimately enhancing patient results, and to assess the clinical outcomes and prognosis of dengue fever patients with cardiac involvement during hospitalization and follow-up visits.

## Materials and methods

A comprehensive longitudinal investigation took place at the Department of General Medicine, a tertiary healthcare facility located in South Gujarat. This study spanned from May 2021 to October 2021. A sample size of 104 cases was determined, based on an expected 14.9% prevalence of cardiac involvement in dengue fever cases [11], with a 95% confidence level, 80% power, and 7.5% absolute error. The sampling technique involved the inclusion of 104 eligible dengue fever cases who were selected on a first come first serve basis after the beginning of the study. Patients were selected based on specific inclusion and exclusion criteria.

Inclusion criteria included dengue fever cases in individuals aged 18 years and above, with either nonstructural protein 1 (NS-1) antigen or immunoglobulin M (IgM) antibody positive or both positive.

Exclusion criteria comprised a previous history of any cardiac disease, history of chronic kidney disease (CKD), diabetes mellitus (DM), and hypertension (HTN), as well as admission ECG suggestive of old ischemic changes, and use of medications affecting heart rate.

Confirmed cases of dengue fever meeting the inclusion criteria were enrolled, and data on socio-demographic profiles and investigations were collected and entered into Excel 2019 (Microsoft, Redmond, WA, USA). Statistical analysis was performed using OpenEpi 3.0. Categorical data were presented as numbers (percent) and compared among groups using the Chi-square test, while continuous data were presented as mean and standard deviation and compared using the student's t-test. A probability (P-value) less than 0.05 was considered statistically significant to draw inferences based on the study's aims and objectives.

The Human Research Ethics Committee, Government Medical College, Surat issued approval number GMCS/ETHICS/Approval/10565/20.

## Results

A longitudinal study conducted at a hospital included 104 confirmed cases of dengue patients. The study revealed that the average age of these patients was 28.6 years, accompanied by a standard deviation of 8.6 years. The ratio of males to females in the study population was 3:1.

Out of 104 patients, the most prominent age group involved was 23-27 years, with the highest percentage of male patients (84%) and a smaller female representation (16%). Conversely, the 18-22 age group had a comparatively balanced gender distribution, but the lowest overall patient count. The 28-32 and >33 years age groups demonstrated somewhat similar gender distributions (Table 1).

**Table 1:** Distribution of Dengue Patients (n=104) by Age Group and Gender n=Number, %

Age Group (years)	Male n (%)	Female n (%)	Total n (%)
18-22	19 (65.5)	10 (34.5)	29 (27.9)
23-27	21 (84)	4 (16)	25 (24)
28-32	20 (76.9)	6 (23.1)	26 (25)
>33	17 (70.8)	7 (29.2)	24 (23.1)
Total	77 (74)	27 (26)	104 (100)

Clinical manifestations suggesting cardiac involvement in dengue are diverse and include chest pain, palpitations, irregularities of pulse, bradycardia, hypotension, and pulmonary edema. The prevalence of symptomatic cardiac involvement among a group of individuals categorized by gender is shown in Table 2. Among the male participants, 27.3% (21), and among the female participants, 72.7% (56), displayed symptomatic cardiac involvement. Considering the entire cohort, 26.9% (28) showed symptomatic cardiac involvement, while 73.1% (76) did not (Table 2).

**Table 2:** Symptomatic Distribution of Cardiac Involvement in Dengue Cases (n=104) n=Number, %

Cardiac Involvement	Male n (%)	Female n (%)	Total n (%)
Present	21 (27.3)	07 (26)	28 (26.9)
Absent	56 (72.7)	20 (74)	76 (73.1)
Total	77 (74)	27 (26)	104 (100)

We focused on the creatine kinase-MB (CK-MB) levels across two distinct categories: "Normal" and "Elevated". The analysis encompassed measurements taken on both Day 1 and Day 3. In the "Normal" group, which consisted of 65 patients, the mean CK-MB level was 20.51 U/L on Day 1, accompanied by a standard deviation of 3.56. The associated p-value for the Day 1 measurement amounted to 0.0001, indicating a statistically significant divergence from the reference value. By Day 3, the mean CK-MB level declined to 18.18 U/L, with a standard deviation of 3.11, and the p-value for this day was computed as 0.0003, underscoring a noteworthy alteration from the Day 1 value.

Conversely, the "Elevated" group encompassed 39 patients, exhibiting a noticeably higher mean CK-MB level of 93.59 U/L on Day 1, alongside a standard deviation of 11.918. The p-value for Day 1 in this group was not explicitly provided. On Day 3, the mean CK-MB level dropped to 83.97 U/L, with a standard deviation of 12.66. The p-value for Day 3 within the "Elevated" group remains unspecified.

The t-test analysis reveals significant fluctuations in CK-MB levels within both the "Normal" and "Elevated" groups between Day 1 and Day 3. The "Normal" group displayed a reduction in CK-MB levels, while the "Elevated" group experienced a decrease from initially elevated levels (Table 3).

**Table 3:** Comparative Analysis of Creatine Kinase-MB (CK-MB) Levels in Patients on Day 1 and Day 3: Normal vs. Elevated Groups Mean, SD, p<0.05 is considered statistically statistically significant.

CK-MB (U/L) [day1&day3]	Number of patients	Mean [day1]	Standard deviation [day1]	p-Value [day1]	Mean [day3]	Standard Deviation [day3]	p-Value [day3]
Normal	65	20.51	3.56	0.0001	18.18	3.11	0.0003
Elevated	39	93.59	11.918		83.97	12.66	

**Figure 1:**
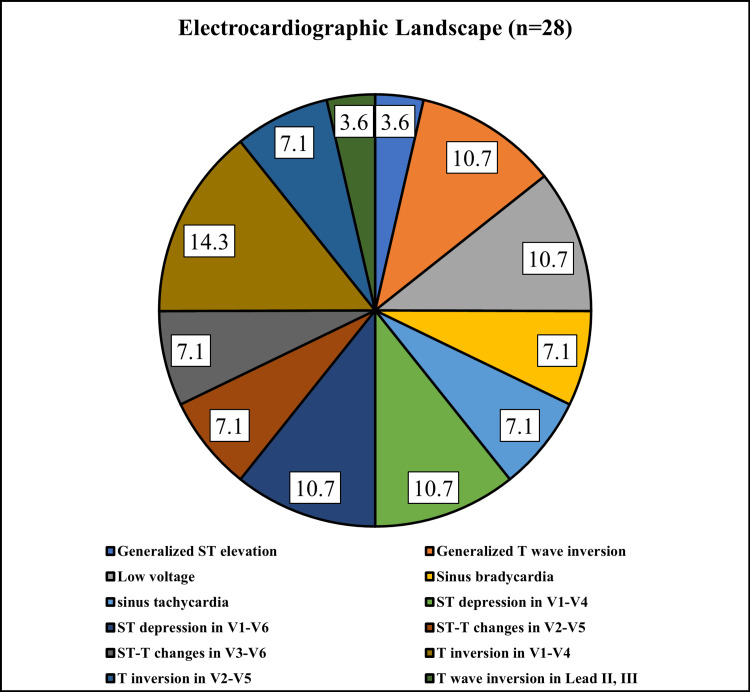
Electrocardiographic Landscape: Unveiling the Diverse Spectrum of Cardiac Changes in Dengue Patients (n=28)

From 104 patients only 28 showed abnormal ECGS. Among the ECG changes observed in the study population that is prolonged QRS interval, QT prolongation, ventricular arrhythmias, and conduction defects, the most common pattern was T inversion in V1-V4, which was present in 14.3% of the cases. This particular ECG alteration showed a relatively higher occurrence compared to other changes. On the other hand, diffuse ST elevation and T wave inversion in Lead II and III were the least common changes, each occurring in only 3.6% of the cases (Figure 1). 

**Figure 2:**
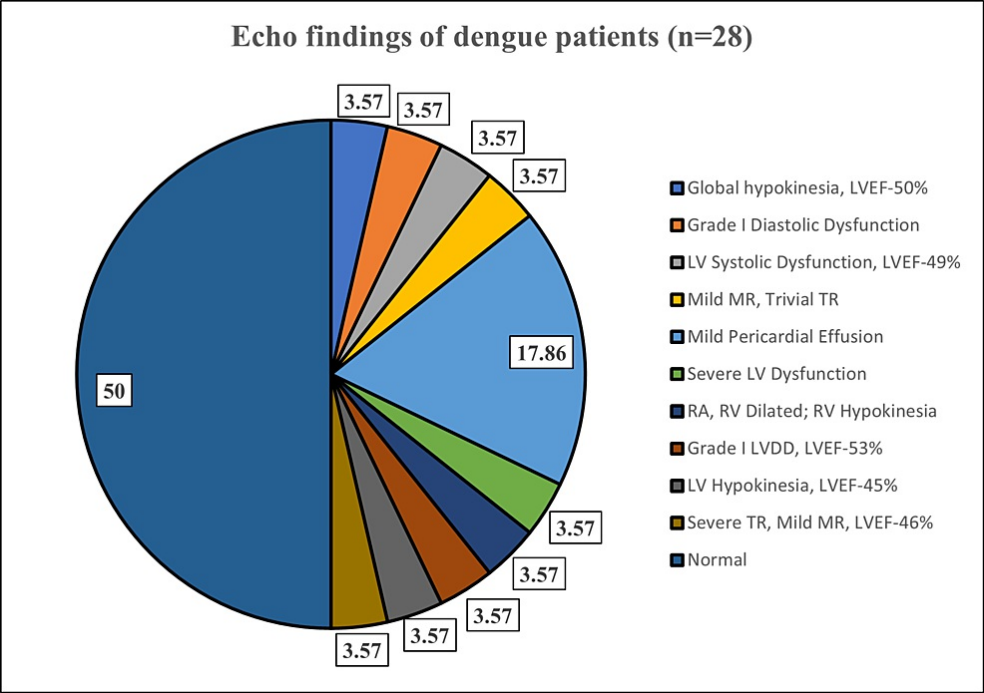
Harmonizing ECHO Discoveries: Unraveling Cardiac Involvement (n=14) ECHO: echocardiography, LV: left ventricle, RV: right ventricle, RA, right atrium, LVEF: LV ejection fraction, LVDD: LV diastolic dysfunction, MR: mitral regurgitation, TR: tricuspid regurgitation

Among the 28 individuals exhibiting ECG changes in dengue fever, only 14 individuals displayed ECHO abnormalities. Among these, the prevailing discovery was a mild pericardial effusion which was seen in five individuals. Other alterations were observed in just a single individual each, indicating that pericardial effusion stands as the primary cardiac anomaly observed in dengue patients (Figure 2).

Overall, the study's findings underscore the prevalence of cardiac involvement in dengue patients, including variations in age groups, CK-MB levels, ECG alterations, and echocardiographic anomalies. Mild pericardial effusion appeared to be a prominent echo abnormality, indicative of the primary cardiac change observed in this population.

## Discussion

Dengue fever has become a notable public health concern in India. Currently, a variety of methods are employed on both the national and state levels to gauge its severity and monitor the occurrence of dengue outbreaks. These approaches are implemented through various National and State Sentinel Surveillance Systems across the country. The National Vector Borne Disease Control Programme and the Integrated Disease Surveillance Programme (IDSP) work together in a passive sentinel surveillance endeavor, focused on the prevention and management of dengue [12].

This study adopted a hospital-based longitudinal approach, enrolling 104 patients with dengue fever who were admitted at New Civil Hospital, Surat, Gujarat. The study aims to uncover potential cardiac involvement in these patients. Inclusion criteria were guided by WHO Criteria-10 for dengue diagnosis, requiring serology (IgM antibody and NS-1 Ag) and aligning with pre-established inclusion and exclusion conditions.

Among the 104 patients, males accounted for 77 (74%), while females constituted 27 (26%). The mean age, represented by mean ± standard deviation, was 29.12 ± 0.92 years. The age spectrum ranged from 19, the youngest patient, to 53, the oldest.

Research carried out in India by Agarwal et al., Ray et al., and Wali et al. individually demonstrated a greater occurrence of dengue infection among male patients in comparison to females. The male-to-female sex ratios reported were 1.9:1, 1:0.57, and 2.5:1, respectively [13-15].

The consistently higher incidence of dengue infection among males in studies conducted could be attributed to factors such as increased outdoor exposure, occupational risks, cultural practices, and potential gender-based immune response differences.

Our study revealed that approximately a quarter of the patients (26.9%) exhibited symptomatic cardiac involvement, with slightly higher rates in males (27.3%) than in females (26%). CK-MB levels showed significant reductions in the "Normal" group from Day 1 to Day 3, while the "Elevated" group experienced a decrease in levels as well. Samples of the troponin from people who have cardiac symptoms were taken from the study population along with CK-MB. However, all the samples had troponin levels less than 0.04 showing no association with the symptoms and hence proving that there was no significance with the cardiac involvement in dengue. However, CK-MB showed fluctuation with symptoms and hence CK-MB proved to have having greater association with cardiac symptoms in dengue.

In 28 patients out of 104, ECG changes included T inversion in V1-V4 as the most common alteration (14.3%), while generalized ST elevation and T wave inversion in Lead II and III were the least common (3.6% each). Among 28 patients with ECG changes, 14 individuals displayed echo abnormalities, primarily characterized by mild pericardial effusion (five individuals). This study underscores the prevalence of cardiac involvement in dengue patients, revealing distinct trends in gender, CK-MB levels, ECG alterations, and ECHO anomalies.

In a study by Salgado et al., which included 102 patients with DF, it was discovered that 14% of these patients showed signs of myocarditis, with a higher prevalence observed among those diagnosed with DHF [16]. This is in line with our study's discovery of 27% cardiac involvement among dengue fever patients. Possible reasons for this correlation could include the sample size of the study population being almost equally distributed.

An investigation conducted in southern India, focusing on cardiac manifestations in a cohort of 100 dengue-infected patients, did not identify any individuals displaying echocardiographic indications of myocarditis [17].

Miranda et al. documented an uncommon and unusual manifestation of dengue illness in which a patient experienced a severe and lethal cardiogenic shock due to acute myocarditis and pericardial effusion leading to tamponade [18]. However, our study did not identify any patients with indications of cardiac tamponade.

Previously conducted studies on patients with dengue infection have consistently highlighted ECG alterations as prevalent cardiac abnormalities. Sinus bradycardia has consistently emerged as the most frequent abnormality in ECG findings [17,19]. Additional ECG abnormalities encompass heart rate and rhythm variations, heart block, waveform anomalies, and voltage irregularities. Our study findings are consistent with these observations, as we also identified T wave inversion followed by sinus bradycardia as the predominant cardiac anomaly among dengue patients exhibiting cardiac involvement.

Arora et al. conducted a study where they identified elevated troponin I levels in serum among 26.7% of patients, and increased CK-MB levels in serum were detected in 33.3% of the cases [20]. Our study similarly found that 37.7 % of patients had elevated CK-MB levels, demonstrating a noteworthy alignment with the previous study's outcomes. This underscores the significance of CK-MB as a vital biomarker associated with cardiac involvement.

Echocardiography holds a vital role in ruling out cardiac conditions that are not related to inflammation, such as valvular diseases, and in monitoring changes in cardiac chamber sizes, wall thickness, ventricular function, and the presence of pericardial effusion. Myocarditis can display itself as overall ventricular impairment, irregularities in regional wall motion, and diastolic dysfunction while maintaining a normal ejection fraction.

Yacoub et al. observed that individuals experiencing severe dengue infection display more notable left ventricular (LV) systolic dysfunction compared to those with mild dengue fever [21]. Our study, wherein only 14 patients displayed ECHO abnormalities, does not align with this observation, considering that the majority of our dengue patients presented with mild dengue fever.

To summarize, our investigation underscores the frequent occurrence of cardiac involvement in individuals with dengue fever. The most prevalent irregularities manifest in the electrocardiogram. The substantial patient population assessed enhances the significance of our insights into the range of cardiac manifestations associated with dengue infection. Notably, our findings indicate that individuals showcasing normal echocardiograms, electrocardiograms, and cardiac marker levels exhibit a lower mortality risk, potentially warranting reduced monitoring or therapeutic intervention.

Limitations

Several limitations are present within this study. The reliance on a single-center methodology could restrict the applicability of the results to more extensive demographics. Additionally, the absence of extended follow-up data and the lack of a control group for comparative purposes introduce constraints on establishing conclusive connections between cardiac engagement and dengue fever as causal relationships.

## Conclusions

Our extensive study of 104 confirmed dengue cases found significant links between dengue infection and cardiac engagement, unveiling uncommon cardiac manifestations beyond the typical symptoms. The study's male predominance (74%) aligns with prior Indian research, possibly reflecting gender-related factors. Notably, the 23-27 age group dominated, urging age-targeted strategies. ECG irregularities, particularly T wave inversion in V1-V4 and sinus bradycardia, highlighted the cardiac impact. Elevated CK-MB levels emerged as consistent cardiac markers, reinforcing their role in assessing severity. ECHO findings, like pericardial effusion, underscore cardiac concerns. In conclusion, our study elucidates various significant findings that advocate for tailored interventions, heightened awareness of cardiac involvement, and strategic use of diagnostic tools. Furthermore, future research should focus on conducting multicenter studies with larger sample sizes and diverse populations to validate the findings of this study.

For recommendations at the individual level, healthcare professionals should be vigilant for cardiac involvement in dengue fever patients, particularly those showing risk factors such as elevated CK-MB levels, ECG abnormalities (e.g., sinus bradycardia), and ECHO abnormalities (e.g., pericardial effusion). Regular monitoring and timely intervention can be crucial in managing cardiac complications and reducing mortality. At the community level, public health awareness campaigns should emphasize the potential cardiac implications of dengue fever, encouraging individuals to seek medical attention if they experience symptoms suggestive of cardiac involvement. Education about preventive measures to control dengue transmission can also indirectly contribute to reducing associated cardiac complications. At the policymaker level, healthcare policies should consider integrating cardiac screening as a routine part of dengue management protocols. Additionally, collaborations between infectious disease specialists, cardiologists, and public health authorities can lead to more comprehensive approaches for managing dengue-related cardiac complications.
